# Novel Data Mining Methodology for Healthcare Applied to a New Model to Diagnose Metabolic Syndrome without a Blood Test

**DOI:** 10.3390/diagnostics9040192

**Published:** 2019-11-15

**Authors:** Mauricio Barrios, Miguel Jimeno, Pedro Villalba, Edgar Navarro

**Affiliations:** 1 Mechatronics Engineering Department, Universidad Autónoma del Caribe, Barranquill 080001, Colombia; 2 Systems Engineering Department, Universidad del Norte, Barranquilla 080001, Colombia; majimeno@uninorte.edu.co; 3 Medicine Department, Universidad del Norte, Barranquilla 080001, Colombia; villalbap@uninorte.edu.co; 4 Public Health, Universidad del Norte, Barranquilla 080001, Colombia; enavarro@uninorte.edu.co

**Keywords:** design methodology, SEMMA, random subsampling, holdout, artificial neural networks, decision tree, principal component logistic regression, metabolic syndrome, diabetes mellitus, heart disease

## Abstract

Metabolic Syndrome (MetS) is a cluster of risk factors that increase the likelihood of heart disease and diabetes mellitus. It is crucial to get diagnosed with time to take preventive measures, especially for patients in locations without proper access to laboratories and medical consultations. This work presented a new methodology to diagnose diseases using data mining that documents all the phases thoroughly for further improvement of the resulting models. We used the methodology to create a new model to diagnose the syndrome without using biochemical variables. We compared similar classification models, using their reported variables and previously obtained data from a study in Colombia. We built a new model and compared it to previous models using the holdout, and random subsampling validation methods to get performance evaluation indicators between the models. Our resulting ANN model used three hidden layers and only Hip Circumference, dichotomous Waist Circumference, and dichotomous blood pressure variables. It gave an Area Under Curve (AUC) of 87.75% by the IDF and 85.12% by HMS MetS diagnosis criteria, higher than previous models. Thanks to our new methodology, diagnosis models can be thoroughly documented for appropriate future comparisons, thus benefiting the diagnosis of the studied diseases.

## 1. Introduction

Machine learning classification models have been used for the diagnosis of several diseases and conditions [1,2]. One type of classification model is to study the probability of a diagnosis of Metabolic Syndrome (MetS). MetS is a group of alterations in metabolism that includes dyslipidemia (abnormal concentrations of blood lipids: increased triglycerides and decreased HDL cholesterol), hypertension, hyperglycemia, and obesity [3]. These are known as metabolic risk factors that increase the likelihood of heart disease or diabetes mellitus [4] since the syndrome indicates a 5-fold increase in the risk of type 2 diabetes mellitus (T2D). Thus, it is often diagnosed as prediabetes [5,6]. Also, the syndrome doubles the risk of developing Cardiovascular Disease (CVD) [7,8]. Other authors relate MetS with the occurrence of cancers and chronic kidney disease [9,10]. Therefore, it is vital to develop mechanisms to achieve the identification of the MetS early in order to avoid or delay its appearance already mentioned by many authors of the scientific community [6,11,12].

The prevalence of the syndrome in countries such as the United States has increased; three studies have yielded the following results: 23.7% in 2002, 34.2% in 2006 and nearly 35% of all U.S. adults were estimated to have the Metabolic Syndrome in 2011–2012 [13] being this last period an estimation of 50% diagnosed MetS in adults mayor of 60 years of age [13]. These studies even showed that the prevalence of MetS is higher among the Mexican-American population. In Mexico has a 41% (95% CI 0.34–0.47) prevalence in adults [14], and in some Latin American countries such as Colombia, several studies on the prevalence of the syndrome were performed focusing on specific populations. For example, a brief study of 62 people in a poor area of Barranquilla, Colombia, found that subjects with arterial hypertension showed a very high prevalence level (74.2%) of MetS based on the ATP III criteria [15,16].

Metabolic Syndrome is diagnosed with a set of risk factors whith some threshold levels in the criteria proposed by several medical associations being recently changed as shown in the Table 1. Some associations proposing criteria are the World Health Organization (WHO) [17], Adult Treatment Panel of the National Cholesterol Education Program (ATP III) [18] , European Group for the study of Insulin Resistance (EGIR) [19] and International Diabetes Federation (IDF) [20].

Since 2009, specialists have had the consensus of Harmonized Diagnosis (HMS), to unify the diagnosis of the syndrome [21]. For diagnosing, MetS must have at least three of the five conditions of the risk factors, except IDF, which requires the central obesity and any two of the four risk factors leftover as shown in Table 1 and emulated using the algorithm shown in Appendixes Appendix A and Appendix B to diagnostic with IDF [22] and HMS criteria, respectively.

The diagnosis always relies on five factors, independent from the criteria used. Of the five elements, two (Waist Circumference (WC) and Blood Pressure (BP)), are obtained in a medical consultation. The other three factors (triglycerides, HDL-C, and glycemia) require invasive tests to know their value in the patient’s blood. The complete diagnosis implies a second medical visit because it is necessary to wait for the test results. The time duration of the syndrome diagnosis goes from the blood test authorization until the arrival of test results which can be days or weeks depending on the health systems [23,24]. For patients at a high risk, the wait time is aggravated due to the occurrence of heart illnesses and diabetes mellitus [12]. In healthcare systems such as the one in Colombia, patients might not return to consultation for multiple reasons, including distance to hospitals, lack of enough providers, among other reasons, thus closing the possibility of starting the appropriate treatment.

The classification models of Metabolic Syndrome without the need of taking a blood sample are essential for the community. Bypassing a blood exam is possible by using variables that can be obtained in a first-level medical care consultation at places such as a community health center. The medical staff can then follow up with a patient with some risk factors of having the syndrome and, in consequence, a high probability of developing diabetes or heart disease at a lower cost because it is a computational model and can be implemented on a large scale easily. Moreover, no clinical exams will be performed to follow up, but only for patients that require a blood test to confirm a pathology.

Therefore, this article details the main classification models of Metabolic Syndrome avoiding/bypassing a blood sample and using variables obtained in a first-level medical care consultation and published in the literature. As the main contributors to this research, we propose a methodology for the diagnosis of similar health conditions and a novel model. These models are compared with the classification model proposed by the authors against data obtained from a study of Metabolic Syndrome performed on the Atlantic coast of Colombia in Barranquilla. We now present the methodology, followed by an application to create a new model for the Metabolic Syndrome, and its results.

## 2. Methodology to Build Data Mining Models

The proposed methodology looks into creating a research tool for research projects solving health-related problems with the help of classification models. Such projects choose their predictor variables based on the relationship between the data and the outcome variable, then compare their results with the results from other authors. The methodology helps the research community by building a classification model from the variables proposed in the literature and comparing the proposals using data from a particular study on the disease. This approach would contribute towards the generalization of a classification model that can be implemented on a large scale like some similar to the Framingham model [25], given that that model has constantly been adding new information to improve the original models, thus taking a circular approach. Therefore, we expect that RAMAD methodology can be further used to get a generalized model to diagnose related diseases.

**Figure 1:**
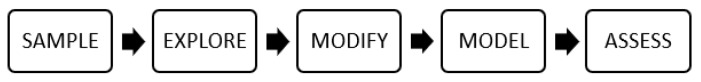
Phase of the SEMMA methodology [26,27].

Many research projects that apply data mining techniques to predict diseases do not explicitly use a methodology but follow a similar approach to those found in the literature. The SEMMA methodology is the most similar to the ones used in those articles. SEMMA is the acronym for the five phases shown in Figure 1 and is promoted by SAS Institute Inc. [26,27]. Many articles explore the data and in some cases, modify it with the creation of new variables to model and evaluate their ability to diagnose. Figure 1 shows the SEMMA methodology, and the phases are explained next.

- **Sample** This step consists of sampling the data by extracting a dataset big enough to contain meaningful information, and small enough to be processed quickly.
- **Explore** the data by searching for anticipated relationships, unanticipated trends, and anomalies to gain understanding and ideas.
- **Modify** the data by creating, selecting, and transforming the variables to then focus on the model selection process.
- **Model** This step consists of modeling data to find data combinations or patterns that reliably predict the desired outcome.
- **Assess** the data by evaluating the usefulness and reliability of the findings from the data mining process [28].

In data mining, there are several methodologies such as KDD (Knowledge Discovery in Databases) and CRISP-DM (Cross Industry Standard Process for Data Mining) [29,30]. However, these are not explicitly used in the analyzed articles because usually authors look for a technique goal, and they do not have a clear business goal as expected [31,32].

**Figure 2:**
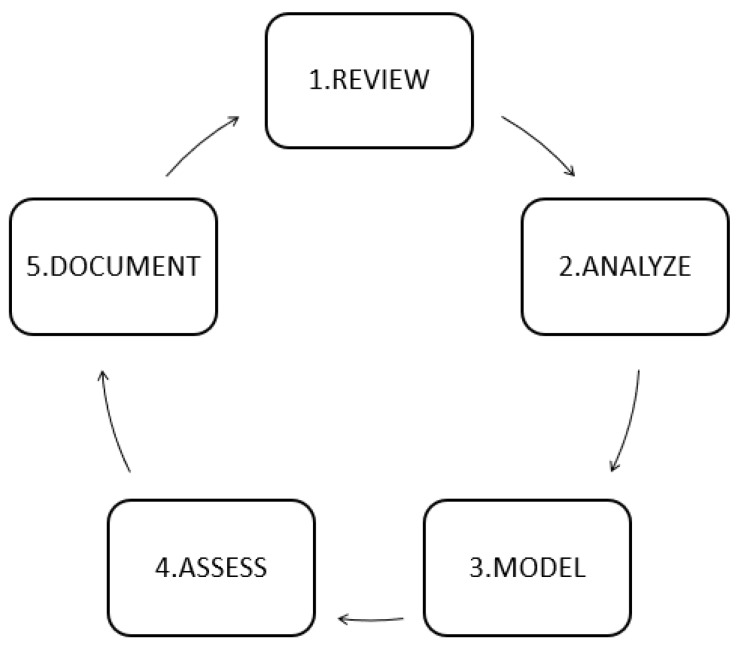
RAMAD methodology.

The methodology proposed in this paper is different from SEMMA as it proposes an incremental circular approach for continuously checking for new models in the literature. Those models are used to compare with the researchers’ data to improve the models based on the newly found information with each new iteration of the methodology, allowing for the reuse of the model with other data. Figure 2 shows the proposed methodology, which consists of the following phases: Review, Analyze, Model, Assess, Document (RAMAD).

1. REVIEW: This phase should start with a research question for the studied disease. Then, the user of the methodology performs a literature review that collects and revises the works proposing classification (diagnosis) models for the disease. It should also describe how to obtain the data of the target population, which could come from publicly available data or data obtained as part of the project.
2. ANALYZE: It is a data analysis that includes a data description, correlation between data, and other tests.
3. MODEL: It consists of building several classification models and testing them using the obtained data. If it is possible, propose a classifier model to detail which models are better used to predict the disease.
4. ASSESS: In this phase, researchers must assess the proposed models and perform a comparison between them using the selected performance indicators.
5. DOCUMENT: Record the parameters of the models and verify that they are detailed so that the project and experiments can be replicated.

The execution of the methodology can be updated and is encouraged by starting again in the Review phase and adding new models from the literature. This circular approach should ensure that researchers keep updating original models, and with appropriate documentation, new researchers could contribute to an improved diagnosis model.

## 3. Application of the Methodology to Metabolic Syndrome

The previously explained methodology is used for Metabolic Syndrome. As stated in the review phase, after defining the disease to be studied, the researchers need to establish a research objective or question to help define the search criteria during this phase. In this exercise, the question is whether we can diagnose the syndrome without using variables taken from invasive tests. From this point, the review phase starts.

### 3.1. Review

**Figure 3:**
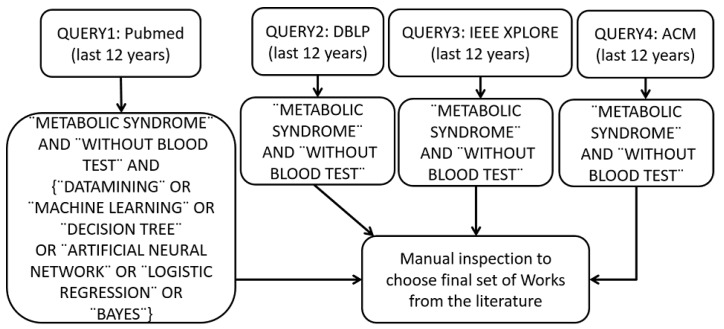
Selection criteria for choosing classification models for MetS diagnosis from the literature.

Literature review follows the approach proposed by [33,34,35] and it is described in the Figure 3. The search criteria includes the topic of data mining techniques applied to the disease. In this work, the studied disease is Metabolic Syndrome, and we are interested in models which do not use a blood sample. It was made using four search engines: DBLP, IEEE XPLORE, ACM and PubMed with a window of 12 years counted retroactively from 2019.

The keywords used in the search process are Metabolic Syndrome, without a blood test, Data mining, and Machine learning due to the similarities associated with data mining. To overcome this difference, a search for articles in PubMed (QUERY1) was carried out with keywords "METABOLIC SYNDROME" AND “WITHOUT BLOOD TEST” AND (“DATA MININ” OR “MACHINE LEARNING” OR “DECISION TREE” OR “ARTIFICIAL NEURAL NETWORK” OR “LOGISTIC REGRESSION” OR “BAYES”). Moreover, in DBLP, IEEE XPLORE, and ACM we searched for “METABOLIC SYNDROME” AND “WITHOUT BLOOD TEST” (QUERY2), (QUERY3) and (QUERY4) without mentioning “data mining” and “machine learning” since these keywords would generate too many results. Manual inspection was performed and then filtered by the criteria established as shown in Table 2.

The results of the queries (QUERY1: 101, QUERY2: 51, QUERY3: 90 and QUERY4: 81) provided a large list of articles and conferences. However, not all the documents had a direct relationship with the Metabolic Syndrome since DBLP, IEEE XPLORE and ACM delivered articles not only of data mining and machine learning but of articles of computer science at a general level. Then the delivered list was filtered, evaluating its relationship with the syndrome, and also excluded repeated articles (mirror articles). The manual inspection documented in Table 2, which reduced the list to a collection of 4 articles, gives the specific requirements to be included in the final list. Each article was evaluated based on the established criteria. These articles used classification models for the diagnosis of Metabolic Syndrome without taking a blood sample using variables obtained in a medical consultation, as shown on the Table 3. The table shows the variables and if the authors used them explicitly in their model, or implicitly.

Kroon et al. [36] proposed a decision tree model to eliminate the need for blood tests for the diagnosis of the MetS in 50–90% of the cases. The  variables used were BMI, WCD, SBP, BPD, and in an implicit way, SEX, and WC due to thresholds of WC and also SBP and DBP. The authors conducted this study with 642 young adults between 17–28 years old. Diagnosis of MetS was defined according to NCEP ATP III with a sample that only had 7.48% of the dataset with MetS (48/642). It is important to remark that subjects with BMI ≥ 35 all had MetS. This paper was the first to use a machine learning model in the problem of diagnosis of the MetS without biochemical variables. However, the authors did not report the model’s performance measure variables used in their dataset.

Romero et al. [37] build several tools to predict MetS in young Mexicans using BMI, WC, Weight, Height, Sex, and other non anthropometrics variables. However, it does not take into account the variables of systolic and diastolic pressure which are risk factors for the diagnosis of MetS. They configured an Artificial Neural Network and trained it with diagnosed patients using the HMS criteria with 70%. They then tested the model using 30% of the set, using a total of 826 people. They used the Positive Predictive Value (PPV) as a performance indicator which varies from 38.2% to 45.4%. Also, the authors used a particular ANN of 25 hidden layers using BMI, WC, Weight, Height, and Sex getting an average PPV 38.8 with a standard deviation 12.8.

Chen et al. [38] proposed a neural network to diagnose the Metabolic Syndrome without using biochemical variables such as blood glucose and cholesterol levels. The authors proposed instead the use of anthropometric variables such as Sex, Age, BMI, WC, HC, WHR, SBP, and DBP, and in an implicit way, they used Weight and Height to measure the occurrence of the Metabolic Syndrome. They compared the technique of Principal Component Logistic Regression (PCLR) with neural networks for predicting Met with criteria IDF.

Hsiung et al. [39] through a sample of 154 subjects with a prevalence 40.26% of MetS segmented in four groups depending on the number of factors of the criteria for the metabolic syndrome with criteria ATP III with variables from physical evaluation, lifestyle profile, heart rate variability, and blood analysis. After excluding the invasive blood tests; the results of multivariate logistic regression identified like non-invasive evaluation variables (blood pressure, body mass index and very lower frequency of heart rate variability) that were significant predictors for the risk of suffering from the metabolic syndrome. This work is noteworthy because it relates the characteristics of the variability of the heart rate with the Metabolic Syndrome as well as the variable Neck Circumference (NC). However, the authors did consider this variable, which can not be obtained with equipment that is in the first level of medical attention. Therefore, this work is excluded from the analysis of the comparisons with the other models.

Kupusinac et al. [22] developed an Artificial Neural Network (ANN) to predict MetS that includes non-invasive variables that easily can be obtained by applying low-cost diagnostic methods. The ANN entry vectors are sex, age, BMI, WHR, SBP, DBP, and in an implicit way WC, Weight, Height due to the use of WSR and BMI. The ANN output is dichotomous in true/false for the prediction of the diagnosis of the MetS with criteria IDF. The ANN training, validation, and testing are carried out in the large dataset that includes 2,928 people, and the authors built a Feed-forward ANNs with 1–100 hidden neurons. The solution achieved the highest positive predictive value PPV = 0.8579 and a Negative Predictive Value NPV = 0.8319.

An important point to highlight is the methodology used by most authors, which is similar to SEMMA. However, they do not mention a specific data mining methodology in their articles. Therefore, the similarities of the phases of these articles with the phases of SEMMA are due to its development. The articles worked by finding the best data mining technique to diagnose MetS without explaining in detail their knowledge about the syndrome or the application, that said, without considering the business objectives [40]. Moreover, none of the articles worked by contributing to existing models to advance in the search for a generalized model with data from multiple populations. Such type of approach could contribute to more robust models. The absence of collaborative work is where the idea of the RAMAD methodology comes into play. Although it does not focus on collaborative work, it encourages a circular review and analysis process, with appropriate documentation. Our methodology aims at building an excellent classification model for the diagnosis of diseases using results obtained by the scientific community. This approach promotes continuous improvement through a circular and incremental methodology.

### 3.2. Analyze

The data used in this paper comes from a study performed in the second semester of 2012 by Universidad del Norte. A survey determined the prevalence of Metabolic Syndrome and cardiovascular risk factors in adults of the city of Barranquilla. The study used a sample of 1478 adult subjects 20 years old or older, randomly selected in 10 neighborhoods and distributed proportionally according to the town, neighborhood, and block of residence. Of this group, those who had: diabetes mellitus, hypertension, or obesity, with ages between 20 and 96 years of age were taken into account to enter the study.

The following group of people was not included: those who did not give their consent, and pregnant women. There is another group of people not included in the data. Those are people with physical or mental health disorders, under steroids treatment, and carriers of decompensated thyroid pathology. The research was carried out according to the Good Clinical Practices (GCP) guide and the International Conference on Harmonization (ICH). Therefore, respect for the dignity and the protection of the rights and well-being of people prevailed.

The researchers respected the individuals’ privacy and their autonomy and decision not to participate in the survey. The survey consisted of 190 questions divided into several sections. The most relevant parts are tobacco consumption, physical activity, family history, obesity, alcohol consumption, sedentary behavior associated with screen devices, anthropometric measurements, and biochemical blood measurements.

The project brought together the respondents into the University del Norte’s hospital, where the programmed clinical examination took place, executed by a doctor and a nurse. The research team measured blood pressure with an already calibrated mercury manometer and took two blood doses with an interval of 5 min. The stature was measured using a stadiometer, the weight using an electronic scale, without shoes and with the least amount of clothes possible. They measured the waist and hip circumferences with flexible metric tapes.

From the sample of 1478 previously surveyed people, the study found that 615 (41.61%) had at least one cardiovascular risk factor. From this group, medical personal took biochemical measurements after resting for 10 min. using a venous puncture. The procedure determined the following variables: lipid profile (cholesterol, triglycerides), fasting blood glucose, glycosylated hemoglobin, and serum insulin. For the diagnosis, this work uses two criteria IDF and HMS to compare how that affects the classification’s performance of the model.

### 3.3. Model

**Figure 4:**
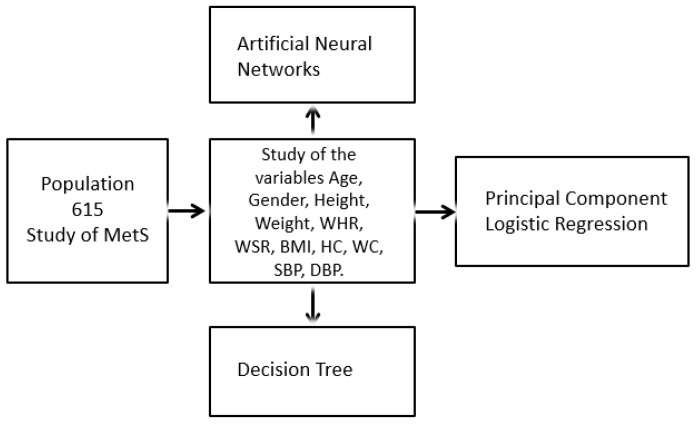
Process of analysis of the classification models.

Figure 4 describes in detail the diagram of the modeling phase. The data is composed of the variables obtained from the state of art resulted of the review stage to be processed using machine learning classification models such as decision tree, principal component logistic regression, artificial neural networks that authors used to diagnose the syndrome without biochemical variables. These techniques are briefly explained now.

#### 3.3.1. Decision Tree

The decision tree classifies data into a set and determines the values that a variable will take from a data entry. It is a process where decisions are made in sequence descending through the tree.

#### 3.3.2. Principal Component Regression Logistic

The dataset is transformed into new variables, which generate the original vectors through a linear combination as shown in the Equation (1) and are selected for their variance (σ≥1).

(1) $$PC_{n}=C_{0}*X_{0}+C_{1}*X_{1}+\ldots \;C_{n}*X_{n}$$

These new variables are orthogonal and apply to the technique of logistic regression that the result of nominal qualitative type depends, or not, on other predictor variables, that is, $PC_{n}$. The nature of the predictor variables can be dichotomous and quantitative. The Equation (2) represents the logistic regression where P is the probability of occurrence of a true positive in Equation (3) and $\beta_{n}$ are the regression coefficients through the Wald statistic.

(2) $$\ln\frac{P}{1-P}=\beta_{0}+\beta_{1}*PC_{1}+\ldots \;\beta_{n}*PC_{n}$$

(3) $$P=\frac{e^{\beta_{0}+\beta_{1}*PC_{1}+\ldots \beta_{n}*PC_{n}}}{1+e^{\beta_{0}+\beta_{1}*PC_{1}+\ldots \;\beta_{n}*PC_{n}}}$$

#### 3.3.3. Artificial Neural Network

**Figure 5:**
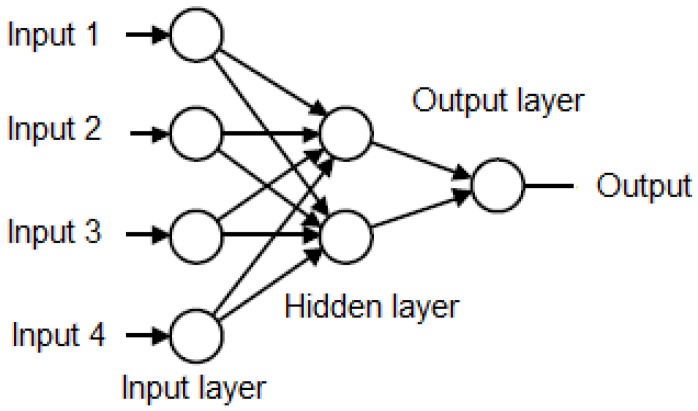
Basic structure of the artificial neural network [41,42].

Artificial neural networks (ANN) is a mathematical representation that models the functioning of a biological neural network to solve problems that require learning and prediction. This type of networks is formed by more than one neuron whose elements are a set of input that can come from other neurons or the outside as shown in Figure 5 the basic structure of ANN.

**Figure 6:**
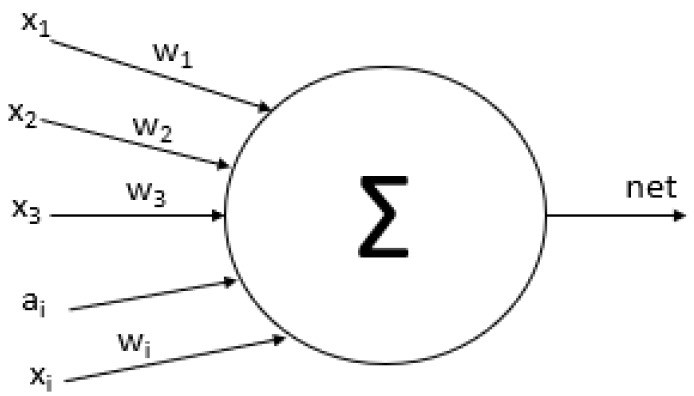
Basic artificial neural.

This structure has several input layer and some hidden layer and one output layer. Each node is an artificial neural has synaptic weights which are the degree of communication between neurons as shown in Figure 6 and the Equation (4). Where $x_{i}^{k}$ are inputs, $w_{i}^{k}$ are synaptic weights, a are bias in the input layer, k is the iteration and n is the number of inputs resulting a net output [42,43].

(4) $$net^{k}=\sum_{i=1}^{n}\left(\omega_{i}^{k}\chi_{i}^{k}-a_{i}^{k}\right)$$

When the net ANN is positive the neuron will be excited and otherwise inhibited. However, if the value is zero, there is no communication between neurons. It also has a propagation rule which integrates the information that comes from different activation function neurons which is responsible for determining the current state and finally converges all the data to the output function [22].

### 3.4. Assess

In this phase, the assessment is carried out using the validation techniques proposed by the authors’ models obtained in the literature review to compare the performance indicators and to deduce the best model for the disease diagnosis.

#### 3.4.1. Hold Out

The original dataset is split into two different datasets labeled as a training and a testing dataset. This can be a 60/40 or 70/30 or 80/20 split with a random distribution.

#### 3.4.2. Random Subsampling

For this validation, multiple data are randomly chosen from the dataset and combined to form a test dataset, that is, multiple holdouts. The remaining data forms the training dataset, and others are tested with a distribution 70/30 or 80/20. The test data predictions give a realistic estimate of the external validation data predictions because of is asymptotically consistent, resulting in more pessimistic predictions of the test data compared to cross-validation [44,45].

#### 3.4.3. Indicators for the Evaluation of Models

To compare the classification models to diagnose the metabolic syndrome without using a blood sample, indicators were used to evaluate their capacity for discrimination, such as Recall, Specificity, Accuracy, Area Under receiver operating characteristic Curves (AUC) [41].

(5) $$Sensitivity\left(SS\right)=\frac{TP}{TP+FN}$$

(6) $$Specificity\left(SP\right)=\frac{TN}{TN+FP}$$

(7) $$PPV=\frac{TP}{TP+FP}$$

(8) $$NPV=\frac{TN}{TN+FN}$$

(9) $$Accuracy\left(ACC\right)=\frac{TP+TN}{TP+TN+FN+FP}$$

(10) AUC=∫(SS)(1-SP)

The values of TP, TN, FP and FN represent True Positives, True Negatives, False Positives and False Negatives respectively. It should be noted that all these models will be specially evaluated using the AUC and the criterion proposed by and Hosmer and Lemeshow [46] that is shown in the Table 4.

### 3.5. Document

Authors using the methodology should write a report or article to give specific and detailed information about the data analysis performed, with a description such as mean and standard deviation by each feature. Such report must also include the parameters of the classification or regression models with their performance indicators and validation methods used for the experiments to allow for replication by other researchers. In the following Results section, we present the analysis and document the whole process in such a way that it can be reused later by other authors to improve the presented model.

## 4. Results

The algorithms of ANN, decision tree, principal component logistic regression were performed and validated using MATLAB as well as the different types of validations such as hold out and random subsampling.

### 4.1. Data Description

The analysis of the data from the patients who were diagnosed based on the IDF and HMS criteria is detailed in Table 5. The data is divided into two populations to explain the prevalence by gender, consisting of a total of 348 women and 267 men. Besides that, from the data, 262 presented a diagnosis of MetS, and 348 were used as control according to IDF. On the other hand, with the HMS criteria, 252 presented a diagnosis of MetS, and 363 were used as a control group, as observed in Table 1. The analysis shows that only ten positive cases (7 men and 3 women) vary between the two criteria showing a possible association. We checked with a Chi2 test resulting in *p* ≤ 0.0001, evidencing a strong association between them. Nevertheless, both criteria were used for modeling.

The age of the subjects is between 20 and 96 years, with an average of 43 years for women and 42 years for men. Also, the prevalence of MetS was 42.6% of the total sample divided into 44.94% among men and 40.8% among women for the criteria IDF and the prevalence of MetS was 40.98% of the total sample divided into 42.32% among men and 39.94% among women for the criteria HMS.

Table 6 shows the statistical description as average(m) and standard deviation (SD) of the variables: Age, Weight, Height, WHR, WSR, BMI, HC, WC, SBP and DBP of each group between healthy people, with MetS, No MetS, and the total. It also showed the Sex variable the number of women and men (women/men) by each group.

### 4.2. Experimenting with Models

This phase conducted experiments using the variables and techniques proposed in the analyzed works by Kroon [36], Ivanovic [22], and Chen [38]. The purpose was to build their performance indicators for the dataset of the population of the Atlantic coast of Colombia.

For the comparison, the experiments used only the data coming from the study of 615 subjects by Universidad del Norte. The variables used to compare were those found in all the articles, which are: Age, Sex, BMI, WC, HC, WSR, WHR, SBP and DBP, and other dichotomic variables. Healthcare professionals can obtain these variables at the first medical consultation.

**Figure 7:**
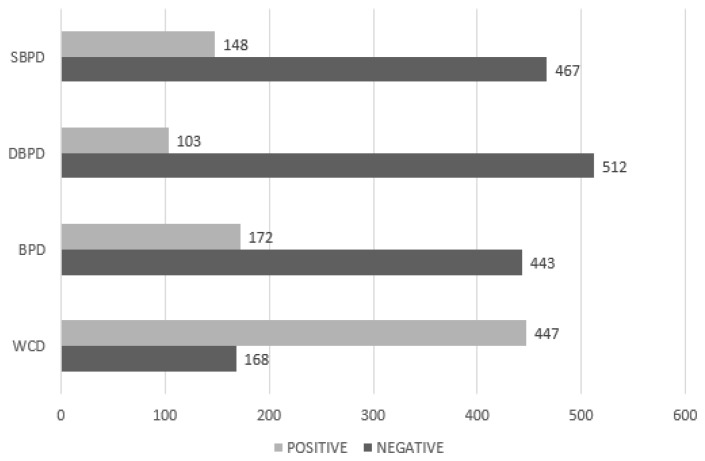
Dichotomous variables.

On the other hand, healthcare professionals take into account the criteria shown in Table 1 for compliance with the risk factors, to diagnose the syndrome. The Waist Circumference (WC) and Blood Pressure (BP) variables are compared with their respective thresholds to become the positive (1) or negative (0) dichotomous value called WCD and BPD that represents the status normal and raised of the values of the waist circumference and arterial pressure, respectively. This approach transforms them into dichotomous variables, which details their values in Figure 7, where the dichotomous variables of systolic (SBPD) and diastolic blood pressure (DBPD) are also shown.

After that, the data was analyzed using the machine learning models proposed by each author. Kroon [36] proposed the regression tree technique, and the output is a dichotomous decision value for MetS. The authors used the BMI variables and two dichotomous variables WCD and BPD. These were used to replicate the experiment using algorithm shown in Appendix C. However, the model proposed by Kroon [36] was built for patients 30 years old or younger. Therefore, the data was fixed to that range of age, resulting in a sensitivity 84.85%, specificity 53.85%, PPV 86.15%, NPV 51.22%, accuracy 77.78% and AUC 68.69% using IDF criteria. On the other hand, for practical purposing, we performed new experiments, but with all the ages, and the results improved the sensitivity of 80.27%, specificity 74,17%, PPV 82.92%, NPV 70,63%, accuracy 77,89% and AUC 76,78% using IDF criteria.

On the other hand, other authors, as [37] proposed an ANN with 25 hidden layers, which has two neurons in the output layer, and the input has five neurons. One neuron by each one variable: WC, Sex, Height, Weight, BMI. This ANN was training using training data (70%) and testing data (30%) obtaining as resulted in a sensitivity 75.25%, specificity 58.62%, PPV 66.97%, NPV 68%, accuracy 67.39% and AUC 76.06% using IDF criteria.

From now on, it will be understood that all the ANN were used with the back-propagation configuration and will always have the same number of neurons as inputs variables in the input layer.

Chen [38] used two data mining techniques to diagnose metabolic syndrome. First, it used Principal Component Logistic Regression(PCLR) with the variables SEX, AGE, BMI, WC, HC, WHR, SBP and DBP where it obtained the principal components of the training data and the testing data getting the following Equations (11)–(13).

(11) $$PC_{1}=-0.142Sex+0.093Age+0.230BMI+0.253WC+0.192HC+0.192WHR+0.178SBP+0.160DBP$$

(12) $$PC_{2}=0.045Sex+0.402Age-0.192BMI-0.255WC-0.184HC-0.199WHR+0.494SBP+0.394DBP$$

(13) $$PC_{3}=0.543Sex+0.241Age+0.278BMI+0.001WC+0.513HC-0.429WHR+0.046SBP-0.207DBP$$

These were used to find the principal components through the 615 patients to test the model of logistic regression shown in the Equation (14) proposed by Chen [38], using the training data.

(14) $$Logit\left(P\right)=-1.809+1.722PC_{1}+0.276PC_{2}+0.403PC_{3}$$

Then, Logit(P) turns into the predictive variable $Y_{p}$ with the Equation (3) mentioned in the Methdology section. So, we were getting performance indicators of sensitivity 59.02%, specificity 0%, PPV 100%, NPV 0%, accuracy 59.02% and AUC 49.86% using IDF criteria.

Chen [38] then used the Equation (15) to normalize the variables and divided the dataset into two: training data (70%) and testing data (30%), to train and test an artificial neural network which has a back-propagation type configuration.

(15) $$X_{N}=\frac{X-Xmin}{Xmax-Xmin}$$

This network has 5 hidden layers with hyperbolic tangent sigmoid function and an output layer with a linear function. It is important to note that Chen [38] did not publish the ANN configuration parameter, only the number of hidden layers and the activation function. Thus, it is necessary to build the ANN with the parameters shown in Table 7. This configuration was set by Kupusinac [22] in its article to diagnose MetS.

Therefore, to replicate the Chen [38] experiment of ANN with 5 hidden layers, we decided to split the data into two parts: the training data (70% of the data) and the testing data (30% of the remaining data). With this step, the analysis shows a performance indicator of sensitivity 76,71%, specificity 71,67%, PPV 84,82%, NPV 59,72%, accuracy 75% and AUC 80,95% using IDF criteria.

Kupusinac [22] proposed an optimization model to find the number of hidden layers between 1 to 100 to obtain the maximum average of PPV and NPV, repeating the tests 100 times with the random subsampling validation and using the 6 variables SEX, AGE, BMI, WSR, SBP, and DBP. This approach creates two configurations of neural networks of 85 hidden layers and 96 hidden layers.

When replicating the experiment, training and testing data are normalized to get the performance indicators with the same proportion of distribution in the data for comparing the performance indicators. After running the experiment, we obtained a mean sensitivity of 73.81%, specificity 65.89%, PPV 77.65%, NPV 60.18%, ACC 70.34%, and AUC 75.8%, for ANN with 85 hidden layers. Moreover, ANN with 96 hidden layers, we obtained a mean sensitivity 74.15%, specificity 66.03%, PPV 77.58%, NPV 60.8%, ACC 70.61% and AUC 76.64%.

To homogenize the experiments using ANN, Chen [10] proposed a network of 5 hidden layers with the random subsampling validation to get the performance indicators of sensitivity 76.47%, specificity 70.22%, PPV 80.41%, NPV 64.31% and AUC 81.75% using IDF criteria and In the same way, for the ANN of [37] with 25 hidden layers, the results showed a sensitivity 72.64%, specificity 63.78%, PPV 76.21%, NPV 58.47%, and AUC 77.13%.

### 4.3. Data Analysis

A correlation analysis was also carried out between the variables that are related to obesity. Table 8 shows that Waist to Hip ratio (WHR) present a very low correlation with all the variables related to obesity. Also, the Waist to Stature Ratio (WSR), Hip Circumference (HC) and Waist Circumference (WC) present a high correlation with body mass index (BMI). This result reaffirms the importance of WC as a parameter to indicate the degree of obesity. It also shows why some criteria recommend it as a priority for the diagnosis of Metabolic Syndrome. Some criteria even consider it as eliminatory because if you do not meet this criterion at the same time, you could not have the syndrome according to the IDF criteria.

After analyzing the correlations of the variables in the dataset and adding the dichotomic variables for the next analysis, we proceeded to select the variables that influence the best in diagnosing the Metabolic Syndrome to reduce the dimensions of the dataset to a subset. For this purpose, we used Sequential Feature Selection searches for a subset of the features in the full model [47] with comparative predictive power being the variables described in Table 6 in conjunction with the dichotomous variables described in Figure 7.

A dataset consisting of the variables HC, WCD, and BPD is obtained and in an implicit way the variable gender because the threshold WC depends on gender. It also performed an analysis of correlation as shown in Table 9, which shows a very low correlation between them.

The selected variables from the dataset were divided into 70% for training and 30% for testing in an ANN. This technique was used due to a higher AUC when compared to the other techniques (decision tree and principal components logistic regression ) to improve the diagnosis rate using only three variables that doctors can get in the first medical consultation and validating with random subsampling.

We chose three (3) hidden layers due to methodology mentioned by [48,49,50] in which they established that the number hidden layers should be 2/3 of input variables plus an output variable resulting in three. This ANN configuration was trained and tested with normalized data to validate through random subsampling to obtain the performance indicators: sensitivity 76.17%, specificity 82.59%, PPV 90.54%, NPV 58.9%, ACC 77.41%, and AUC 87.36%.

As a summary, the performance indicators of each experiment is shown in the Table 10, Table 11, Table 12 and Table 13, where the first two tables show results for the IDF criteria and the next two tables show results for the HMS criteria.

Regardless of the diagnostic criteria, the behavior of data mining techniques shows that ANN of 3 hidden layers is better compared to the previously proposed techniques. It seems that decreasing hidden layers increases AUC.

However, it is important to note that each ANN has a different dataset of variables, and so, these are different each only ANN85, and ANN96 has the same variables.

Table 10, Table 11, Table 12 and Table 13 shows that two techniques or configurations were better in some performance indicators. ANN using 8 variables (AGE, SEX, WHR, BMI, HC, WC, SBP, DBP) with 5 hidden layers, which has a sensitivity of 80.27%, NPV 70.63%, and ACC 77.89% higher when compared with other machine learning models. However, ANN with 3 hidden layers using 3 variables (WCD, DAP, HC) and SEX, which was implied, is the best due to the performance indicator of specificity 82.59%, PPV 90.54% and specially AUC 87.36%.

The previous results allow classifying patients with MetS (i.e., the sensitivity) very selectively. This selective classification is possible because the healthcare professionals always presume that every patient is healthy, and this algorithm can guarantee that when a patient with MetS is identified, it is because they have a high probability (approximately 90.54%) that they suffer from it. Concerning specificity, the ANN obtained 82.59%, being superior to the other models, indicating that the ANN is an excellent tool to rule out the probability of the positive diagnosis of the syndrome, because of every 100 healthy patients, the ANN confirms 83 approximately healthy people.

On the other hand, as regards to AUC, the ANN technique obtained 87.36%, being superior when compared to others. This result means that an individual randomly selected from the group of patients has a test value higher than one randomly selected from the healthy group at 87.36% of the time considered excellent according to the dissertation of Hosmer and Lemeshow [46].

**Figure 8:**
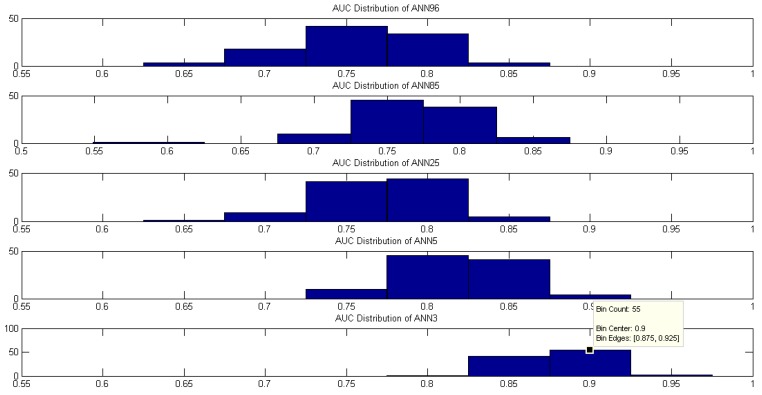
AUC distribution for each ANN to diagnose using the IDF criteria.

**Figure 9:**
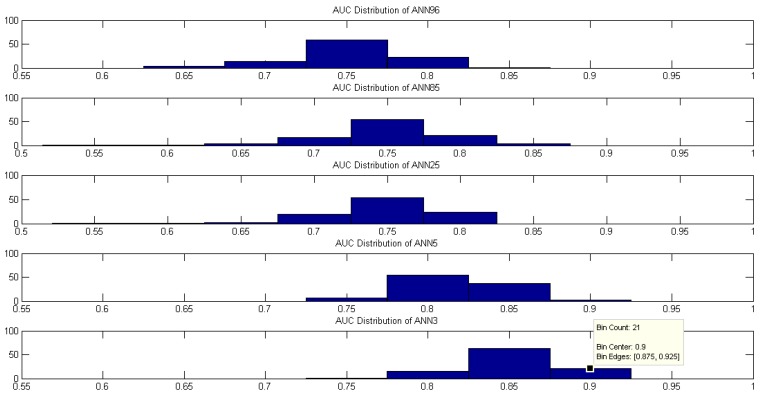
AUC distribution for each ANN to diagnose using the HMS criteria.

The mean was used to quantify the value of the highest probability as IDF as HMS criteria diagnostic because we assume that random subsampling would generate a normal probability distribution. We plotted the distribution of the AUC probabilities, and this was considered as a discriminant value to choose the best classifier among ANN96, ANN85, ANN25, ANN5, and ANN3. Figure 8 and Figure 9 show that the average does not reflect the highest AUC probability rate. The ANN3 has, in its zone of occurrence, higher AUC levels unified in the range between 0.875 to 0.925 with a probability of 55%, and median 87.43% to IDF. The same happens with the HMS criteria, which presents higher AUC levels unified in the range 0.875 to 0.925 with a probability of 22%, and median 85.36%. Hence, HC, BPD, and WCD input variables in ANN with 3 hidden layers have an excellent performance concerning the other proposed models with the dataset collected by Universidad del Norte in Barranquilla.

## 5. Discussion

Metabolic syndrome is a constellation of several risk factors of developing cardiovascular disease and type 2 diabetes that requires a blood test for diagnosis. However, with the technique of data mining, the scientific community has built models to obtain data that can get through first-level medical attention without the need of a blood test. This paper has shown a possible association between IDF and HMS criteria that is checked with a Chi2 test resulting in *p* ≤ 0.0001, evidencing a strong association between them which was explained by the difference of only 10 positive cases (3 women and 7 men) of MetS. Also, according to the review phase of the proposed methodology, the most used criteria in the models used to diagnose MetS without blood test were the IDF and in second place was the ATP III, which have some small differences in the levels of some variables. However, since 2009, the specialists have had the consensus of Harmonized Diagnosis (HMS) to unify the diagnosis of the Metabolic Syndrome, which a previous paper already used [37]. Thus, the models explained used IDF and HMS criteria to compare the results with other future models.

The analyzed learning models allow the diagnosis of the Metabolic Syndrome with an AUC higher than 70% without using a blood sample, early avoiding or delaying its appearance because some risk factors such as obesity can be modified. It is interesting to note that the ANN technique has better precision than PCLR and DT. However, the latter provides rules and equations that researchers can implement at a lower computational cost. They also generate a priori knowledge about the relationships of the non-invasive variables with the Metabolic Syndrome due to their white-box model.

Several models used variables relative to the obesity such as Body Mass Index, Waist Circumference, Hip Circumference, Waist to Hip ratio, and Waist to Stature ratio. During the correlation analysis step of the data analysis phase, we found a good correlation between them except with Waist to Hip ratio. These variables, when processed together, can generate a problem that commonly exists in the medical statistical literature, called multicollinearity. A way to avoid the multicollinearity is to use variable transformation. Waist Circumference WC becomes a Dichotomous variable (WCD) to decrease the correlation with Hip Circumference.

The data indicated that the ANN model of three hidden layers using WCD, BPD, and HC according to its AUC of 87.75% to IDF and 85.12% to HMS are excellent to discriminate the data. The results are excellent in comparison to principal component logistic regression and decision trees and the other ANN model. All were compared based on the biomedical dataset of 615 people of the Atlantico, Colombia region, of whom 42.6% had Metabolic Syndrome.

Therefore, it is recommended to make more comparisons with a larger dataset to strengthen the thesis that the ANN model is superior for the diagnostic of Metabolic Syndrome without using a blood test and so, we only take measurements of triglycerides, HDLC and glycemia in necessary cases. Thus, this project only used variables that can be obtained in a medical consultation at the first level of health care or community health center.

The two decision tree and ANN techniques have in common that they diagnose through three variables of which two are common and are WCD and BPD and demonstrate the importance of dichotomous variables when diagnosing diseases and support why they are recognized as risk factors.

Researchers could replicate the methodology and its application presented in this paper in other areas where data collection from patients is happening. Such projects include those where researchers obtain biomedical data from trials or the immersion of health monitoring sensors in the world (for example, smart city, smart wearables, among others). These applications, together with the technique of data mining, can find patterns to make decisions and find many solutions. Such is the case of the diagnosis and monitoring of pathology development, and in consequence, the improving of the well-being of people and disease prevention with the help of these learning models.

This article proposed a methodology to find a model to diagnose diseases under certain conditions, in this case, is MetS without a blood test using variables obtained in first-level medical attention were the result of the effective ANN model with three hidden layers and only three variables. Therefore, we recommend the methodology to get a model generalized or outstanding model. Furthermore, it is very important and necessary to test with more data from different populations.

RAMAD methodology offers the advantages to a range of entities, such as doctors, patients, health systems and communities. Doctors have the advantage of obtaining the method with better performance to be able to predict diseases that depend on variables obtained with non-invasive procedures, such as the metabolic syndrome.On the other hand, the patients with an early diagnosis can go on with the treatment immediately managing to reduce their risk factors that are related to diabetes and heart disease.

As for the healthcare system, it will be able to do the proper monitoring of diseases in patients who live in remote areas by predicting their status, before the disease becomes intractable and deadly. So, it manages to reduce the cost of health care monitoring and management caused by these diseases and the delaying of the treatment. It can also help generate new knowledge about patients with MetS diagnosed in a non-invasive way by discovering new treatments that, when implemented, will reduce the risk of developing diseases resulting from MetS.

By having RAMAD methodology, the community will be able to acquire new non-invasive diagnostic methods that will allow an increase in the level of health and well-being due to the fact that people will measure the progress of their health status with the help of technological applications that will keep them motivated.

In the very near future, the RAMAD methodology can be improved using more precise methods such as deep learning with a greater number of data captured in real time with the implementation of smartcity and big data where the end user, that is, the patient can use the emerging technologies in the field of health care, clinical score, m-health, e-health [51]. Additionally, the next-generation point of care (POC) technologies will benefit from advances in data mining methodology for the improvement of the parameters of which some are used and the increase in the number of users that require the use of this technology [52,53].

In general, this is a sufficiently detailed study and that reports a novel methodology which can be applied not only to MetS, but also to a variety of other diseases, such as cardiovascular diseases, diabetes, etc. Moreover, this opens the possibility to enable the development of generalized models for such diseases.

## 6. Conclusions

Metabolic syndrome is diagnosed according to the most up-to-date methods, and all healthcare professionals agree on using five criteria, three of which are obtained through blood analysis. Healthcare providers then collect the results and apply the criteria through the decision threshold according to each method. Although using principal component logistic regression, decision trees for the processing of data obtained from non-biochemical variables, it has been proven that metabolic syndrome can be diagnosed with an acceptable discrimination ability because of an AUC ≤ 0.8. Being the learning model of ANN with 3 hidden layers obtained a higher AUC of 87.75% to IDF and an AUC of 85.12% to HMS that are excellent. Therefore, ANN with three hidden layers using WCD, BPD, and HC is an excellent model for identifying metabolic syndrome and helps to reduce the time of treatment initiation, which allows for the development of simple prevention strategies for the subject at risk for type 2 diabetes and cardiovascular disease.

The results of this article reflects the importance of monitoring blood pressure, hip circumference, and waist circumference for diagnosing the metabolic syndrome. The results also reflect the importance of dichotomous variables in medical decision making. Caregivers can obtain these variables through tools usually essential in the first-level medical attention such as a tape measure and a sphygmomanometer.

These results come from the application of the proposed methodology, which highlights the importance of continuously reviewing new findings, applying changes to the model, and adjusting accordingly. Therefore, the contribution of this paper is not only the model but a continuous methodology to get a generalized model using data mining techniques which should attract other researchers to contribute and continue with this work to achieve a generalized model of MetS. Researchers could apply such methodology to other diseases to construct generalized models for those diseases.

In the future, the method can probably be improved with the use of fuzzy logic, the Bayesian network and other data mining techniques. However, it is recommended that healthcare professionals can use the method to help in the treatment and diagnosis of the early signs of diseases such as diabetes or coronary heart diseases related with metabolic syndrome in order to take preventative measures that ensure that these diseases do not develop.

## Figures and Tables

**Table 1:** Diagnosis criteria.

Risk Factors	ATP III(2001)	IDF(2006) and HMS(2009)
Central Obesity WC Waist Circumference	Male: WC ≥ 102 cm Female: WC ≥ 88 cm	Country/ethnic,specific values for WC South American population Male: WC ≥ 90 cm Female: WC ≥ 80 cm
TG Triglycerides	>150 mg/dL	>150 mg/dL or treatment
FG Fasting Glucose	>100 mg/dL without diabetes	>100 mg/dL or treatment
HDL-C High-density lipoprotein cholesterol	Male:<40 mg/dL Female:<50 mg/dL or treatment	Male:<40 mg/dL Female:<50 mg/dL or treatment
BP, SBP, DBP Blood Pressure Systole and Diastole Blood Pressure	SBP ≥ 130 mmHg and DBP ≥ 85 mmHg or treatment	SBP ≥ 130 mmHg or DBP ≥ 85 mmHg or treatment

**Table 2:** Criteria established for choosing the articles.

Criteria for the Selection of Articles
Can be obtained the variables through first-level medical attention? Y/N
What diagnostic criteria do authors use? e.g., ATP II, IDF, HMS or other criteria recognized.
What datamining technique do authors use? e.g., logistic regression, decision tree, artificial neural networks, and others
What validation method do authors use? e.g., hold out, random subsampling and others.
What performance indicators do authors use? e.g., Area under the ROC curve, sensitivity, specificity, positive and negative predicative values and accuracy

**Table 3:** Variables and technique used by the authors.

Authors	Kroon et al. [36]	Romero et al. [37]	Chen et al. [38]	Kupusinac et al. [22]
Age			E	E
Sex	I	E	E	E
Weight	I	E	I	I
Height	I	E	I	I
WHR: Waist to Hip ratio			E	
WSR: Waist to Stature ratio				E
BMI: Body mass index	E	E	E	E
HC: Hip circumference			E	
WC: Waist circumference	I	E	E	I
WCD: WC Dichotomous	E			
BPD: Blood Pressure Dichotomous	E			
SBP: Systolic blood pressure	I		E	E
DBP: Diastolic blood pressure	I		E	E
Technique	DT	ANN	PCLR, ANN	ANN

DT:Decision Tree; PCLR: Principal Component Logistic Regression; ANN: Artificial neural networks; E: Explicit use of variable; I: Implicit use of variables

**Table 4:** Assessment rules of AUC [38].

AUC	Discrimination Ability
AUC=0.5	No discrimination
0.5 < AUC < 0.7	Regular
0.7 ≤ AUC < 0.8	Acceptable
0.8 ≤ AUC < 0.9	Excellent
AUC ≥ 0.9	Outstanding

**Table 5:** Relationship between IDF and HMS in the database.

Criteria	HMS		IDF	
Gender	No MetS	MetS	No MetS	MetS
Men	154	113	147	120
Women	209	139	206	142
Total	363	252	353	262

**Table 6:** Statistic description of the total data of the study variables.

Variables	MetS	No MetS	Total
AGE m(SD)	47.62(17.49)	38.89(15.96)	42.61(17.17)
WC m(SD)	99.81(11.33)	87.24(11.91)	92.59(13.21)
HC: m(SD)	105.51(10.56)	93.73(12.50)	98.75(13.07)
BMI: m(SD)	29.09(5.31)	25.26(4.74)	26.89(5.33)
WHR: m(SD)	0.94(0.05)	0.93(0.09)	0.94(0.08)
WSR: m(SD)	0.61(0.67)	53.79(7.43)	0.56(0.79)
SBP: m(SD)	128,52(18,46)	112,91(12,61)	119.55(17.19)
DBP: m(SD)	78.48(11.13)	71.18(9.21)	74.29(10.69)
SEX (Women/Men)	(142/120)	(206/147)	(348/267)

Average(m); Standard deviation (SD); Women/Men.

**Table 7:** Parameter of the ANN [22].

Parameter	Value
Training Function	Levenberg-Marquardt backpropagation
min_grad	10${}^{-10}$
mu	10${}^{-3}$
mu_dec	0.1
mu_inc	10
mu_max	10${}^{10}$
HL function	hyperbolic tangent sigmoid
Out function	linear

**Table 8:** Correlations between obesity related variables of Universidad del Norte data.

	BMI	WC	HC	WSR	WHR
BMI	1.00	0.78	0.79	0.79	0.06
WC	0.78	1	0.86	0.92	0.32
HC	0.79	0.86	1	0.84	−0.20
WSR	0.79	0.92	0.84	1	0.21
WHR	0.06	0.32	−0.20	0.21	1

**Table 9:** Correlation among HC, BPD and WCD.

	HC	BPD	WCD
HC	1.0000	0.3015	0.6459
BPD	0.3015	1.0000	0.2275
WCD	0.6459	0.2275	1.0000

**Table 10:** Performance indicator versus technique using hold out validation with IDF criteria.

	DT [36]	ANN25 [37]	PCLR [38]	ANN5 [38]
SS	80.27%	75.25%	59.02%	76.71%
SP	74.17%	58.62%	0%	71.67%
PPV	82.92%	66.97%	100%	84.82%
NPV	70.63%	68%	0%	59.72%
ACC	77.89%	67.39%	59.02%	75%
AUC	76.78%	76.06%	49.86%	80.95%

**Table 11:** Performance indicator versus technique using random subsampling validation with IDF criteria.

	ANN96 [22]	ANN85 [22]	ANN25 [37]	ANN5 [38]	ANN3
SS	74.15%	73.81%	72.64%	76.47%	76.39%
SP	66.03%	65.89%	63.78%	70.22%	82.52%
PPV	77.58%	77.65%	76.21%	80.41%	90.24%
NPV	60.8%	60.18%	58.47%	64.31%	59.46%
ACC	70.61%	70.34%	68.79%	73.7%	77.46%
AUC	76.04%	75.8%	77.13%	81.75%	87.75%

**Table 12:** Performance indicator versus technique using hold out validation with HMS criteria.

	DT [36]	ANN25 [37]	PCLR [38]	ANN5 [38]
SP	74.17%	59.46%	0%	68.52%
PPV	82.44%	71.96%	100%	84.40%
NPV	67.94%	57.14%	0%	49.33%
ACC	76.26%	65.76%	57.40%	70.11%
AUC	75.19%	75.48%	50.19%	78.97%

**Table 13:** Performance indicator versus technique using random subsampling validation with HMS criteria.

	ANN96 [22]	ANN85 [22]	ANN25 [37]	ANN5 [38]	ANN3
SS	71.62%	72.22%	69.44%	75.37%	75.08%
SP	66.95%	66.25%	63.73%	72.54%	80.15%
PPV	77.4%	76.05%	75.74%	81.38%	87.17%
NPV	58.67%	60.80%	55.34%	64.21%	59.94%
ACC	69.33%	69.42%	68.88%	73.91%	75.35%
AUC	74.94%	74.79%	74.8%	81.74%	85.13%

## Appendix A

**Algorithm A1** Algorithm to diagnostic MetS with IDF criteria.
1: Hit=0; 2: DxMetSIDF=0; 3: **if** ((SEX==0)and(WC≥80))or((SEX==1)and(WC≥90)) **then** 4: Hit=1; 5: **if** (SBP≥130)or(DBP≥85) **then** 6: Hit=Hit+1; 7: **end if** 8: **if** ((SEX==0)and(HDLC<50))or((SEX==1)and(HDLC<40)) **then** 9: Hit=Hit+1; 10: **end if** 11: **if** TG≥150 **then** 12: Hit=Hit+1; 13: **end if** 14: **if** (GLU≥100) **then** 15: Hit=Hit+1; 16: **end if** 17: **if** (Hit≥3) **then** 18: DxMetSIDF=1; 19: **end if** 20: DxMetSIDF=0; 21: **end if**

## Appendix B

**Algorithm A2** Algorithm to diagnostic MetS with HMS criteria.
1: Hit=0; 2: DxMetSHMS=0; 3: **if** ((SEX==0)and(WC≥80))or((SEX==1)and(WC≥90)) **then** 4: Hit=Hit+1; 5: **end if** 6: **if** (SBP≥130)or(DBP≥85) **then** 7: Hit=Hit+1; 8: **end if** 9: **if** ((SEX==0)and(HDLC<50))or((SEX==1)and(HDLC<40)) **then** 10: Hit=Hit+1; 11: **end if** 12: **if** TG≥150 **then** 13: Hit=Hit+1; 14: **end if** 15: **if** (GLU≥100) **then** 16: Hit=Hit+1; 17: **end if** 18: **if** (Hit≥3) **then** 19: DxMetSHMS=1; 20: **else** 21: DxMetSHMS=0; 22: **end if**

## Appendix C

**Algorithm A3** Algorithm to diagnostic MetS risk with ATP III criteria using the decision tree [36].
1: **if** BMI≥35 **then** 2: DxMetS=1; 3: **else** 4: **if** (BMI<35)and(BMI≥30) **then** 5: **if** (WCD)and(BPD) **then** 6: DxMetS=1; 7: **else** 8: **if** (WCD)or(BPD) **then** 9: DxMetS=1; 10: **else** 11: **if** (WCD)and(BPD) **then** 12: DxMetS=0; 13: **end if** 14: **end if** 15: **end if** 16: **else** 17: **if** (BMI<35)and(BMI≥30) **then** 18: **if** (WCD)and(BPD) **then** 19: DxMetS=1; 20: **else** 21: **if** (WCD)or(BPD) **then** 22: DxMetS=1; 23: **else** 24: **if** (WCD)and(BPD) **then** 25: DxMetS=0; 26: **else** 27: DxMetS=0; 28: **end if** 29: **end if** 30: **end if** 31: **end if** 32: **end if** 33: **end if**
